# Comments on the optimal use of medical ozone in clinics versus the Ozone High Dose Therapy (OHT) approach

**DOI:** 10.1186/s41231-022-00132-6

**Published:** 2022-12-05

**Authors:** Marianno Franzini, Luigi Valdenassi, Sergio Pandolfi, Giovanni Ricevuti, Umberto Tirelli, Francesco Vaiano, Salvatore Chirumbolo

**Affiliations:** 1 International Scientific Society of Oxygen Ozone Therapy (SIOOT), Gorle, BG Italy; 2 Department of Drug Science, University of Pavia, Pavia, Italy; 3 Tirelli Clinical Group, Pordenone, Italy; 4 Department of Neurosciences, Biomedicine and Movement Sciences, Unit of Human Anatomy, University of Verona, Strada Le Grazie 8, 37134 Verona, Italy

## Background

The recent paper by König and Lahodny reported that a therapeutic approach on their own, accounting on the use of high doses of ozone (140,000 μg) via major autohemotherapy, modulates some fundamental mitochondrial parameters towards an improvement in their functionality [1]. The report left us completely dumbfounded and somehow disappointed.

Fundamentally, it is widely known that ozone is a toxic compound, able to increase the oxidative stress and to damage mitochondria [2], so the recent paper by König and Lahodny should raise some critical concern in an expert reader [1].

Few years ago, Bocci stated that ozone can be either a toxic or a medically useful compound [3], and actually, the use of ozone as a therapeutic approach accounts on this apparently paradoxical hallmark [3–6]. The puzzling behavior attributed to ozone is grounded on the fine use of ozone dosages. Ozone acts in a hormetic way. Relatively low doses of ozone in the blood, usually by activating the release of low dosed lipo-peroxides, such as 4-HNE, are able to promote the role of reactive oxygen species (ROS) as signaling molecules [7]. When used as signals, ROS activate mitochondria biogenesis and organelle’s activity, decrease the impact of inflammation and pyroptosis and stimulate cytoskeletal organization, so contributing in maintaining the optimal activity of the survival machinery of the cell [7–9]. According to Bocci, the ozone entering the bloodstream via a classical oxygen-ozone major autohemotherapy (O_2_-O_3_-MAHT), including a dose range very close to 30–50 μg/ml (extended range = 10–80 μg/ml), when blood is exposed to ozone for very few minutes, will reach a dose range between 0.21 and 1.68 mM, quite far from the genotoxic effects attributed to ozone 5 mM in saline solution [10–12]. Noticeably, 0.21 mmol/L is the concentration (10 μg/ml) used to demonstrate the ability of ozone to improve mitochondria activity in vitro [9].

Notwithstanding, despite this evidence, according to the authors the use of Ozone High Dose Therapy (OHT) ameliorated mitochondria function in a group of patients recurring to the clinic [1]. As the authors used a complessive ozone amount at least 15 times higher than usual O_2_-O_3_-MAHT, we wondered how they reached the reported results.

A first look on the patients’ recruitment showed no eligible criteria reported. The authors selected six patients, who were scheduled for the Ozone High dose Therapy (OHT) protocol, in order to withdraw the peripheral blood mononuclear cell (PBMC) fraction, for the in vitro investigation of mitochondria function. There is no information about how the patients were selected, a heterogeneous clinical setting (4 patients lacking of any acute or chronic illness) characterized their access to the study. PBMC withdrawn from venipuncture peripheral blood specimens were cultivated and investigated in vitro for the mitochondria functional parameters a) basal oxygen consumption rate (OCR), b) ATP-linked OCR and proton leak, c) maximal OCR and reserve capacity and d) non-mitochondrial OCR [1]. The Bioenergetic Health Index (BHI), the maximal Oxygen Consumption Rate (OCR), the reserve capacity OCR and the non-mitochondrial OCR, increased significantly following at least 2 sessions of OHT. Finally, the authors reported that OHT improved mitochondria health in patients’ PBMCs [1].

## Methodological issue 1: lacking of clinical impact

A first concern is that their conclusions cannot say anything about the therapeutic meaning of OHT, as the authors limited to assess that OHT improves mitochondria function in PBMCs, so practically investigating a simple in vitro model [1].

Moreover, they introduced an amazing hypothesis, fairly speculative: if OHT improves mitochondria functional parameters and mitochondria health is a foundation for a proper ozone-mediated therapy effect, then OHT might be considered a good alternative to the usual oxygen-ozone autohemotherapy (O_2_-O_3_-MAHT) with relatively low doses of ozone [1]. This might theoretically represent a good reasoning but lacks in vivo evidence and shows some rationale bias.

As a matter of fact, their ozone high dose (OHT) approach, despite starting from six recruited patients, described an in vitro system, targeting mitochondria bioactivity and never translating their evidence to any clinical outcome. If correctly performed, this would have ensured the reader about the clinical effectiveness of OHT and moreover the authors would have described the therapeutic potential of OHT [1].

On the other hand, the authors launched OHT as a true novelty in the field of ozone therapy, as usually the ozone concentration used to treat several ailments and pathologies ranges from 30 to 50 μg/ml, with a total ozone administration in a single oxygen-ozone major autohemotherapy (O_2_-O_3_-MAHT) of about 8.0–9.0 mg/ml (i.e. approximately 11.97–13.46% of O_3_ in O_2_ by weight), considering that the optimal O_3_ concentration is 40–45 μg/ml [3–7, 13–16], but this novelty lacks of clinical evidence.

## Methodological issue 2: the effect of huge doses of ozone on PBMC mitochondria

The authors used a complessive amount of 14,000 μg O_3_ (14 mg O_3_) for each single pass, about 1.55–1.75 times the dosage usually employed by O_2_-O_3_-MAHT and recommended by the International Scientific Society of Oxygen Ozone Therapy (SIOOT) particularly in COVID-19 [17, 18]. The complessive amount of O_3_ in the SIOOT-recommended O_2_-O_3_-MAHT does nor exceed 40,000 ppm, disposed into a maximum of four sessions [18, 19], whereas OHT uses an amount of O_3_ more than three times higher [1]. Yet, these considerations may appear pleonastic, as the paper refers to in vitro evidence [1].

A dose of ozone as low as 0.9–5.3 mmol/L (i.e. 43–254 μg/ml) has been reported as noxious for peripheral blood leukocytes in vitro [11]. Some authors, using either 20 μg/ml or 80 μg/ml O_3_, investigated the effect of ozone on in vitro PBMCs, reporting that 80 μg/ml increased the PBMC release of LDH by 79% and that increased the mitochondrial NADH/NAD^+^ ratio of 40% and, in the presence of oxygen, 80 μg/ml decreased mitochondria respiration (substrate succinate) by 89% and (substrate glutamate and malate) by 80% [20]. This should suggest that a dose higher than 80 μg/ml O_3_ is concerning for mitochondria functionality in PBMCs.

Furthermore, the results plotted by the authors [1], do not have a correspondent list of data in a Table, in order to be aware of the evidence reported and Cohen’s d for BHI parameters were particularly high. Aside from a scant description of mitochondria methods, data do not appear so convincing to an expert reader.

## Methodological issue 3: mitochondria parameters

The use of certain parameters from the authors [1], for example the oxidative consumption rate (OCR), which should represent the whole sum of the cellular processes leading to a net O_2_ consuming, including mitochondria, are particularly clumsy. OCR is not simply a mitochondria parameter, as it includes the activity of other cell oxidases, yet the authors calculated the basal OCR, the non-mitochondrial component and the ATP-linked/proton leak component of OCR, in order to overview the complete mitochondria stress [1]. This kind of test, i.e. the mitochondrial stress test, used in the real time analysis of viable cells, allows researchers to realize about critical respiratory defects. A classical model is to stabilize the basal OCR before adding a sequential order of oligomycin, carbonyl cyanide-4 (trifluoromethoxy) phenylhydrazone (FCCP), which is a mitochondrial uncoupling agent able to collapse the mitochondria proton gradient, disrupting the mitochondrial membrane potential and finally antimycin A or rotenone. The time course will allow to measure the contribution of the non-respiratory chain to O_2_ consumption, its ATP-linked consumption, the maximum reached by OCR following FCCP and the reserve capacity of the cell [21]. Briefly speaking, the authors used the bioenergetic health index (BHI) to investigate the effect of ozone on cells [1, 22].

In our opinion, the equivocal interpretation of the authors about the positive reading of BHI, by their OCR parameters, depends on the ability of O_3_-induced ROS to activate mitophagy [21], which subsequently enhances mitochondria turnover and organelles healthy function [23]. Yet, mitochondria autophagy, via the activation of the PINK1/Parkin signaling pathway, induces the activation of NLRP3 inflammasome and then pyroptosis [24].

Therefore, although high doses of ozone, triggering a ROS-dependent mitophagy should accelerate the mitochondria fission, autophagy and subsequent organelles biogenesis, the mechanism involves a huge pro-inflammatory and pro-apoptotic response, which undoubtedly is a source of concern.

## Methodological issue 4: bias on autohemotherapy

As concerning the application of OHT in clinics, we wondered about a series of statements and data reported by the authors [1], we would like to address further.

The authors reported that they used a L1 (10 passes) Hyper Medozon device (Herrmann Apparatebau GmbH, Im Honing 3, 63,820 Eisenfeld, Germany) and that an oxygen-ozone mixture was generated at an O_3_ concentration of 70 μg/ml [1]. This device, according to the technical specification, can produce a maximum of 4.8 g O_3_/hour, quite far from the Medical 98 HCPS from Multiossigen S.p.A. Gorle (BG), Italy, with a maximum of 60 g O_3_/hour, suggesting that the ozone-producing device from the authors [1] may be not so particularly suited to reach the standards declared. At these ranges, the device reported from the authors should produce 80,000 ppm O_3_, clearly far from 140,000 ppm (140,000 μg/ml) reported in the study.

The use of a positive pressure raised further concerns, moreover. Our group recently investigated the effect of the speed in the reinfusion of the ozonated blood in an O_2_-O_3_-MAHT, by using a near-infrared spectra (NIRS) analysis of the blood flow [25]. During fast reinfusion (80 drops/min) a decrease in oxygenated hemoglobin (O_2_Hb) was observed, whereas during slow reinfusion (50 drops/min) an increase in the vascular bed of microcirculation, with increase in O_2_Hb was reported [25]. Considering that the mean blood pressure is about 127 mmHg (range 115–140 mmHg), i.e. 0.16 atm, and that the normal blood infusion speed is close to 15 drops/min, at 0.9 atm the number of blood drops might be approximately next to 84 drops/min, so falling within the fast reinfusion reported by our studies [1, 25]. Moreover, we seriously wondered if adding oxygen to the reinfusion process at positive pressure does not cause embolism. And finally, adding oxygen during the blood reinfusion process may alter the reactive oxygen species (ROS) balance in the ozone reactivity with blood cells.

## Discussion

König and Lahodny published a paper where they endorsed a new approach of ozone therapy based on high dosage of this oxygen allotrope [1]. Ozone is a toxic xenobiotic for cells and produce toxic lipo-peroxides (LOPs), which only at defined and low dose ranges may exert a beneficial role on cells and organisms [13–16].

The recognized biotoxicity of ozone, opposite to its clinical usefulness [26], compels physicians to use ozone with caution, i.e. to use ozone, usually in a balanced mixture oxygen-ozone, by following a stringent range of concentrations in order to reach a therapeutic outcome and reduce greatly any adverse effect. Actually, as ozone, from a simple chemical point of view, is a toxic xenobiotic, its ability in eliciting a beneficial action in the cell depends on a complex machinery of signaling systems and pathways, which altogether give rise to the mechanism of “hormesis” [26].

Fundamentally, the pharmacological or simply biochemical dose-response associated with a toxicant, which, for example, inhibits a targeted function, should provide an increasing dose-response curve of functional inhibition, whereas, if hormesis occurs, at a certain dose of the dose-response curve, the toxicant reduces its inhibitory effect, paradoxically passing to a stimulatory one, to then resume its inhibitory behavior from a certain dose onward [27–30]. A typical hormetic curve is therefore “U-shaped” [28].

Our experience with SIOOT strongly suggests to use medical ozone, as a therapeutic compound, in the hormetic range. The mitochondrial damage by mitophagy might even “refresh” mitochondria biogenesis but rapidly activates damage signals and the NLRP3-mediated inflammation. If this occur in a chronic inflammatory disease, ozone may worsen rather than ameliorate the patient’s clinical outcome. Furthermore, the many bias in the ozone dosage and way of autohemotherapy let us to believe that OHT may cause failure or even damage to the treated patient, a concern which has to be read as an alarming warning for ozone therapy approaches.

## Conclusions and future remarks

**Fig. 1:**
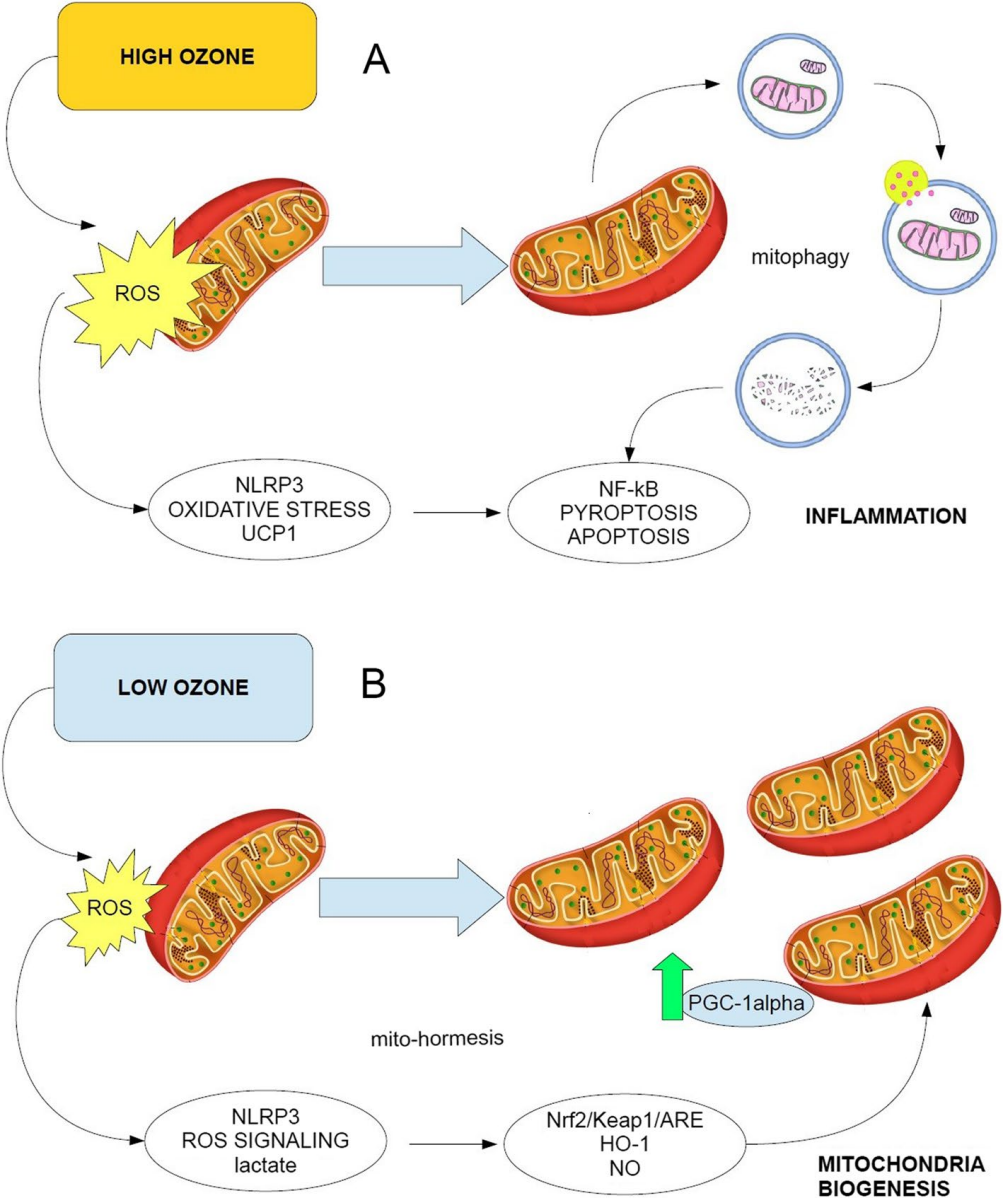
The different action of ozone on mitochondria. **A** High doses of ozone activate a huge ROS response, mitophagy and the shift of the Nrf2/NF-κB rate towards NF-κB, finally leading to an inflamed state even with pyroptosis and apoptosis; **B** Low doses of ozone, slightly activating the NLRP3 via ROS as signaling molecules, increase the rate Nrf2/NF-κB, triggering an anti-oxidant state and inducing mitochondria biogenesis via PGC-1α

Figure 1 summarizes our functional hypothesis about the role of medical ozone in therapy on the basis of existing literature. High doses of ozone activate an immuno-inflammatory response, via a mitophagy mechanism, whereas low doses activate the anti-oxidant system Nrf2/Keap1/ARE and mitochondria biogenesis. König and Lahodny’s paper represents an outstanding example of how empiricism in ozone therapy may raise fundamental concerns in the efficacy and safety of ozone autohemotherapy. The conclusive message that OHT, by improving mitochondria function, is able to successfully treat patients, is biased, as the study does not deal with clinical outcomes and patients’ recruitment was flawed.

We reported elsewhere criticism about how O_2_-O_3_-MAHT should be performed, considering some evidence reported on the field [14, 16]. Safety represents a leading issue in the treatment with ozone, because correct dose, administration route, devices and skilled expertise are altogether paramount for preventing ozone-related adverse effects and lead to a disappointing failure of the O_2_-O_3_-MAHT.

Despite the paper by König and Lahodny would encourage colleagues to deepen the OHT as a possibility, we remain somehow dumbfounded and disappointed about their results, which we consider highly concerning. Yet, we wish them to improve further their research study, in order to dismiss our criticism.

## Data Availability

On request of the Corresponding author.
